# A Rare Presentation of a Rare Disease: Oropharyngeal Dysphagia as The Main Manifestation of Myasthenia Gravis

**DOI:** 10.7759/cureus.16880

**Published:** 2021-08-04

**Authors:** Mohammad Abudalou, Michael Malkowski, Marcel R Robles, Eduardo A Vega, Michael C. C Slama

**Affiliations:** 1 Internal Medicine, St. Elizabeth's Medical Center, Tufts University School of Medicine, Boston, USA; 2 Surgery, St. Elizabeth's Medical Center, Tufts University School of Medicine, Boston, USA; 3 Neurology, St. Elizabeth's Medical Center, Tufts University School of Medicine, Boston, USA

**Keywords:** oropharyngeal dysphagia, myasthenia gravis, autoimmune, deglutition, dysphagia

## Abstract

Oropharyngeal dysphagia is defined as the inability or difficulty to initiate swallowing. It has a wide array of etiologies including structural and neurologic diseases. Myasthenia gravis (MG) is a rare autoimmune condition caused by antibodies against the post-synaptic membranes of the neuromuscular junction, leading to fatigable weakness of skeletal muscles. Bulbar symptoms are less prevalent than ocular symptoms or limb weakness but can be particularly morbid. Non-neurologists are more likely to be the first providers to evaluate patients with dysphagia and should be familiar with MG. We report a unique case of newly diagnosed MG with the initial presentation of solid food and liquid dysphagia.

## Introduction

Dysphagia is a term that describes difficulty in swallowing whether to solid food, liquids, or both. It is comprised of two types including oropharyngeal dysphagia (OD) and esophageal dysphagia. The prevalence of dysphagia varies according to age and health status and ranges between 1.7% and 11.3%, with a higher prevalence in the elderly [1]. OD can lead to severe complications such as aspiration pneumonia and malnutrition [2]. There is a wide array of etiologies that result in OD including structural, neoplastic, inflammatory, central nervous system, and neuromuscular etiologies [3].

Myasthenia gravis (MG) is a rare autoimmune disease that causes fatigable weakness in skeletal muscles. Pathogenesis involves autoantibodies directed against the post-synaptic membrane of the neuromuscular junction. The most commonly encountered autoantibodies are directed against the acetylcholine receptors (AChR); others include antibodies against muscle-specific kinase (MuSK), and even less frequently against low-density lipoprotein receptor-related protein 4 (LRP4). The culprit antibody is not found in some cases (termed seronegative MG). AChR antibodies are of IgG1 and IgG3 subtypes, while MuSK antibodies are of IgG4 subtypes [4]. The annual incidence of MG is estimated between 8 and 30 cases/1 million people. The incidence in the population under the age of 40 years is higher in females, while over 50 years old males are more commonly affected [5-7]. About 12% of patients with MG are found to have a thymoma, but benign thymic abnormalities such as hyperplasia can occur in up to 75% of patients [8]. Symptoms include ocular muscle weakness leading to fluctuating binocular diplopia and ptosis, as well as generalized symptoms due to involvement of limb, axial, bulbar, and respiratory muscles. The majority of patients develop some degree of generalized weakness (generalized MG or gMG) while a minority will have only ocular symptoms (ocular MG or oMG). Diurnal variations and worsening weakness with prolonged muscle use are common, due to impaired neuromuscular junction transmission.

Ocular symptoms are common in myasthenics while bulbar symptoms, such as dysarthria and dysphagia, are less common and are rare as standalone symptoms [9]. gMG with predominantly bulbar weakness can be particularly hard to diagnose for the non-neurologist, as symptoms can be mistaken for other more common medical conditions; yet, this subtype of MG can be particularly morbid if not recognized and treated promptly due to complications from aspiration and respiratory muscle weakness. We present a case of a newly diagnosed myasthenic whose main presenting complaint was oropharyngeal dysphagia.

## Case presentation

A 78-year-old man with a history of prostate cancer, bladder cancer, hypertension, type 2 diabetes mellitus, and chronic kidney disease presented to the hospital with a three-week history of progressive dysphagia. Initially, his dysphagia was mainly to solid foods but progressed to liquids two days prior to presentation. His main symptom was not being able to initiate a swallow, which resulted in solids and liquids getting stuck in the back of his throat. This was associated with difficulty handling his secretions. Associated symptoms in the same time frame included voice hoarseness, generalized weakness, and a 10-pound unintentional weight loss.

Upon arrival, he was hemodynamically stable, with a low-grade fever of 100°F. On physical examination, the patient appeared uncomfortable, with inability to tolerate oral secretions, and evidence of coarse breath sounds diffuse. Neurologic examination was notable for bilateral moderate fatigable ptosis without ophthalmoplegia or report of diplopia; he had nasal and guttural dysarthria, moderate bifacial weakness with cheek puff and eye closure, moderate tongue weakness, mild bilateral upper extremity weakness both proximally and distally, severe proximal lower extremity weakness, and mild distal lower extremity weakness.

**Figure 1:**
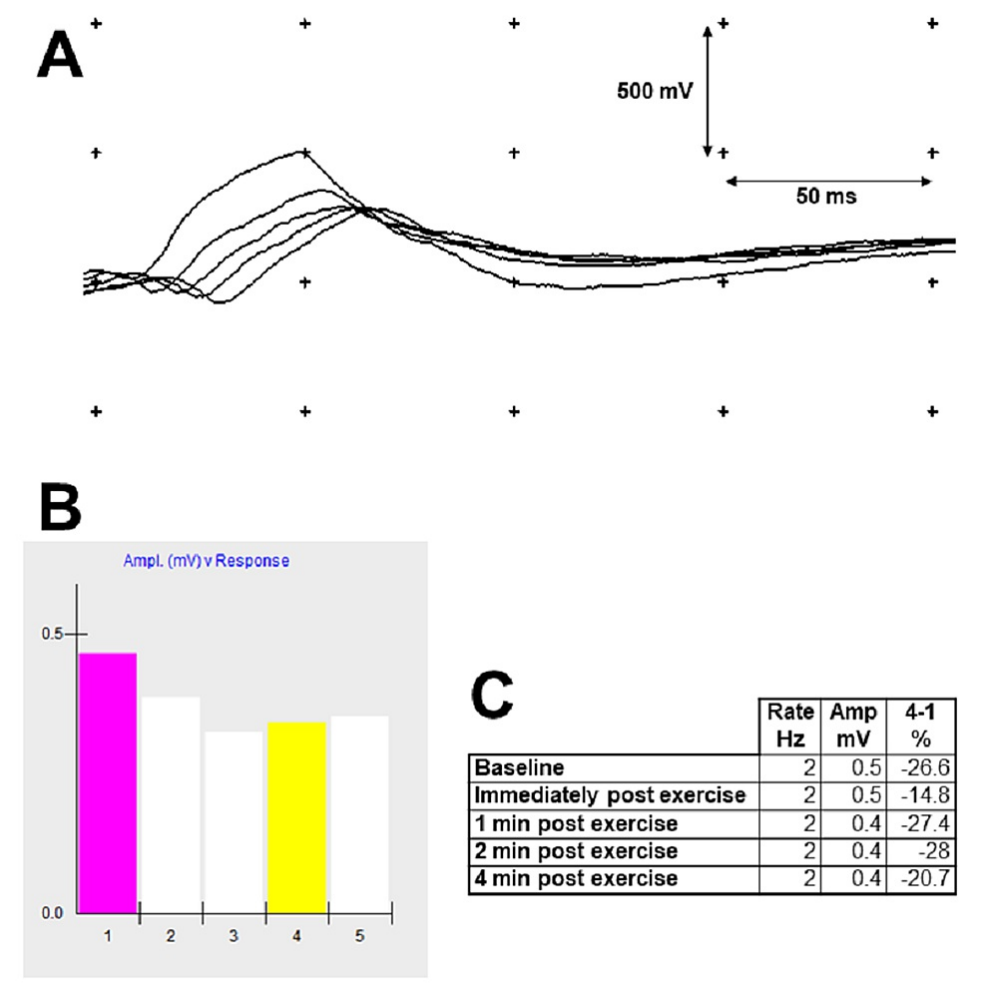
Slow (2 Hz) repetitive stimulation of the right facial nerve recording over the right nasalis muscle showed significant decrement in a pattern consistent with myasthenia gravis (A) Baseline compound muscle action potential response to a 2 Hz stimulation train, showing decremental response amplitude by more than 26% between the first and the fourth potential. A consistent decrement of more than 10% is considered significant and can be suggestive of a neuromuscular junction disorder in the right clinical context. (B) Amplitudes of the baseline responses from (A). There is a partial repair of decrement with the last two responses leading to a characteristic U-shaped decrement that is often seen in myasthenia, due to mobilization of secondary stores of acetylcholine vesicles. (C) Decrement was consistently greater than 10%. There was post-exercise facilitation leading to partial repair of decrement immediately following 15-second exercise of the muscle.

Computed tomography (CT) of the head did not reveal any acute intracranial abnormalities. Electrodiagnostic studies were performed; slow repetitive nerve stimulation of the right facial nerve recording over the right nasalis muscle demonstrated an abnormal decrement in the amplitude of the compound muscle action potentials ranging from 14.8% to 28%, in a pattern consistent with a post-synaptic disorder of neuromuscular junction (Figure 1). Myasthenia gravis was suspected. Computed tomography of the chest showed no thymoma. Negative inspiratory force was 10 cm H_2_O. Pyridostigmine and intravenous immunoglobulin (IVIG) were started but the patient’s respiratory status further deteriorated, and he developed hypercapnic respiratory failure requiring intubation. He received a total of 2 g/kg of IVIG divided over five sessions and prednisone 60 mg daily was added to treatment. His respiratory mechanics progressively improved and he was extubated. Upon extubation, his diet was gradually advanced to a dysphagia pureed diet. His oral secretions lessened, and he appeared more comfortable at rest. Upon discharge to a rehab facility, he was continued on pyridostigmine and prednisone. Mycophenolate mofetil was added as a steroid-sparing agent as an outpatient. A myasthenia gravis laboratory panel that was sent during his inpatient stay eventually returned positive acetylcholine receptor binding (33.3 nmol/L; reference {R}: ≤ 0.30), blocking (50% inhibition; R: < 15%), and modulating antibodies (93% inhibition; R: < 32%). MuSK antibodies were negative.

## Discussion

Deglutition can be divided into four phases, which comprise the oral preparatory phase, oral voluntary phase, pharyngeal phase, and esophageal phase [10]. Disruption of any of these phases can lead to dysphagia. A careful history is imperative to differentiate between oropharyngeal and esophageal dysphagia. Causes of OD are broad and include iatrogenic (medications, post-surgical, radiation, corrosive), infectious (diphtheria, botulism, Lyme disease, syphilis), metabolic (amyloidosis, Cushing’s syndrome, thyrotoxicosis, Wilson’s disease), structural (Zenker’s diverticulum, masses), and neurologic causes. Neurologic causes of dysphagia can localize to the brain (e.g., stroke, inflammatory or mass lesions affecting the brainstem), cranial nerves (e.g., some forms of the Guillain-Barré syndrome, infectious, inflammatory or neoplastic processes affecting the leptomeninges), muscles (e.g., some types of inflammatory myositides such as inclusion body myositis, some forms of genetic myopathies such as oculopharyngeal muscular dystrophy), and the neuromuscular junction (e.g., myasthenia gravis).

Our patient presented with rapidly progressive oropharyngeal dysphagia as his main symptom. His clinical presentation and neurologic examination were most consistent with bulbar-predominant generalized MG. Dysphagia as the main symptom of MG can delay the diagnosis due to the wide differential; as such, a careful review of the system inquiring about other common symptoms of MG, and early neurologic consultation are important. The mechanism behind dysphagia in MG may be due to a coordination disorder between suprahyoid laryngeal elevator muscles and cricopharyngeal muscles of the upper esophageal sphincter. It can be indolent and not clinically apparent due to oropharyngeal compensatory mechanisms [11]. In one study, esophageal manometry performed on 25 myasthenic patients revealed esophageal motor dysfunction without clinical complaints of swallowing disturbance [12].

The diagnosis of MG is supported by several tests. Serologic testing for autoantibodies against AChR, MuSK, and LRP4 are commercially available, but their turnaround time is often several weeks, and a negative serology does not exclude the diagnosis [4]. In the acute setting or seronegative patients, electrodiagnostic studies with slow repetitive nerve stimulation are the main diagnostic modality (Figure 1). In patients with a clinical suspicion but negative serology and negative repetitive nerve stimulation, single fiber electromyography looking for abnormal action potential variability (or “jitter”) can be obtained [13].

The treatment strategy is highly dependent upon the type of myasthenia (ocular only or generalized), the severity of the symptoms, and the type of antibodies present. For ocular myasthenia gravis with no generalized symptoms, pyridostigmine monotherapy is often sufficient. In generalized myasthenics, the mainstay of treatment is pyridostigmine in combination with prednisone; a steroid-sparing agent such as azathioprine or mycophenolate mofetil is often added. IVIG and plasmapheresis are reserved treatments modalities for severe MG [14]. MuSK positive patients typically respond well to plasma exchange but much less so to IVIG. Thymectomy is always indicated in MG patients with thymoma, but may also be beneficial in some patients with generalized MG without thymoma [7]. Rituximab is sometimes used in severe cases and is particularly effective for MuSK positive MG. Eculizumab, a monoclonal antibody that inhibits the complement pathway, has been recently approved for AChR positive generalized MG [15]. Another class of medications, the neonatal Fc (FcRn) receptor antagonists, is a promising avenue for the treatment of generalized MG with several recently completed and ongoing clinical trials [16].

## Conclusions

In summary, we present a case of myasthenia gravis where the primary symptom was rapidly progressive oropharyngeal dysphagia. It is critical to recognize oropharyngeal from esophageal dysphagia to avoid unnecessary endoscopic interventions. Myasthenics whose presenting symptom is oropharyngeal dysphagia are likely to first be evaluated by primary care physicians or gastroenterologists; it is, therefore, crucial for non-neurologists to be familiar with MG and inquire about other common symptoms involving the ocular, bulbar, axial, and appendicular musculature. Early neurological consultation for prompt recognition of this disorder is imperative as delay in treatment can lead to rapid exacerbation and myasthenic crisis. A wide array of immunosuppressive treatments are available for this condition.
